# Total saponin from *Anemone flaccida* Fr. Schmidt abrogates osteoclast differentiation and bone resorption via the inhibition of RANKL-induced NF-κB, JNK and p38 MAPKs activation

**DOI:** 10.1186/s12967-015-0440-1

**Published:** 2015-03-15

**Authors:** Xiangying Kong, Wenbin Wu, Yue Yang, Hongye Wan, Xiaomin Li, Michun Zhong, Hongyan Zhao, Xiaohui Su, Shiwei Jia, Dahong Ju, Na Lin

**Affiliations:** Institute of Chinese Materia Medica, China Academy of Chinese Medical Sciences, No. 16, Nanxiaojie, Dongzhimennei, Beijing, 100700 China; Institute of Basic Theory, China Academy of Chinese Medical Sciences, Beijing, 100700 China; Guangzhou Kanghe Pharmaceutical Limited Company, Guangzhou, 511440 China

**Keywords:** Anemone flaccida, RANKL, Osteoclasts, Bone resorption, MAPKs, NF-κB

## Abstract

Osteoclasts, bone-specialized multinucleated cells, are responsible for bone destructive diseases such as rheumatoid arthritis and osteoporosis. Natural plant-derived products have received substantial attention given their potential therapeutic and preventive activities against bone destructive diseases. In the present study, we investigated the effects of total saponin (TS) from *Anemone flaccida* Fr. Schmidt, on receptor activator of nuclear factor-κB ligand (RANKL)-induced *in vitro* osteoclast differentiation. We observed that TS concentration-dependently inhibited RANKL-induced osteoclast formation from RAW 264.7 cell and bone marrow-derived macrophages (BMMs), as well as decreased extent of actin ring formation and lacunar resorption. The RANKL-stimulated expression of osteoclast-related transcription factors were also diminished by TS. Moreover, TS blocked the RANKL-triggered TRAF6 expression, phosphorylation of mitogen-activated protein kinases (MAPKs) and IκB-α, and inhibited NF-κB p65 DNA binding activity. Furthermore, TS almost abrogated the nuclear factor of activated T cells (NFATc1) and c-Fos expression. Taken together, our results demonstrated that TS suppresses RANKL-induced osteoclast differentiation and inflammatory bone loss via the down-regulation of TRAF6 level, suppression of JNK and p38 MAPKs and NF-κB activation, and subsequent decreased expression of c-Fos and NFATc1. Therefore, TS may be a potential agent and needs to be more evaluated *in vivo* or in clinical trials to become a therapeutic for lytic bone diseases.

**Electronic supplementary material:**

The online version of this article (doi:10.1186/s12967-015-0440-1) contains supplementary material, which is available to authorized users.

## Introduction

Bone remodeling depends on the balance between bone resorption and bone formation [1]. It is necessary to repair damaged bone and to maintain mineral homeostasis. An imbalance in favor of bone resorption, most often due to excess osteoclastic activity, leads to bone loss in pathological conditions such as rheumatoid arthritis (RA) and osteoporosis [2]. Especially focal bone destruction within inflamed joints is the most specific hallmark of RA and leads to deformation, laxity, and functional disability [3].

Osteoclasts are the primary cells responsible for bone resorption and play crucial roles not only in maintaining bone homeostasis but also in development of pathological conditions such as joint destruction in RA [4-6]. Therefore, Osteoclasts become the main targets of current anti-resorptive drugs. The interaction of receptor activator of nuclear factor-κB ligand (RANKL) is essential for osteoclast differentiation and activation [7]. The binding of RANKL and RANK on osteoclast progenitor cells triggers the activation of tumor necrosis factor receptor-associated factor 6 (TRAF6) and subsequently the activation of NF-κB and mitogen-activated protein kinases (MAPKs), such as extracellular signal-regulated kinase 1/2 (ERK1/2), p38 and c-Jun N-terminal kinase (JNK) [8-10]. c-Fos and Nuclear factor of activated T cells (NFATc1) are two important downstream transcription factors in the RANKL/RANK signal pathway and play crucial role on the osteoclast differentiation [9,11].

There is a growing interest in the utilization of medicinal plants for prevention and treatment of bone disorders including RA and osteoporosis [12]. The dry root of *Anemone flaccida* Fr. Schmidt, commonly known as “Di Wu” (Chinese name), is widely used as a folk medicine in the clinical compound prescription for the treatment of rheumatic diseases, external wounds and inflammations in China. Saponins are the characteristic components and also the main active ingredients of *A. flaccida* [13], and Diwu Fengshian Capsule with total saponin (TS) as the main active component has entered phase 3 clinical trial in China [14]. It has also been reported that saponins isolated from *A. flaccida* possess anti-inflammation, immunoregulatory, analgesia and antitumor properties [14,15]. Bone metabolism is tightly associated with immune system and inflammation joint destruction in RA and other forms of arthropathies [16,17]. The anti-inflammation and immune-regulatory properties of TS from *A flaccida* may prove its role in osteolytic bone diseases. However, to date the direct effects of *A. flaccida* on bone metabolism have not been studied.

In the present study, we investigated the direct effect of TS, the major bioactive constituents of *A. flaccida,* on osteoclast differentiation in RANKL-induced RAW 264.7 cells. Moreover, the possible mechanism associated with its inhibitory effect on osteoclast differentiation was also explored.

## Materials and methods

### Chemicals

Alpha Modified Eagles Medium (a-MEM), fetal bovine serum (FBS), L-glutamine, penicillin and streptomycin, were purchased from Invitrogen Life Technologies. Mouse soluble RANKL was obtained from PeproTech Biotechnology. Antibodies against NFATc1 and TRAF6 were purchased from Santa Cruz Biotechnology. Antibodies against c-Fos, ERK, p-ERK, JNK, p-JNK, p38, p-p38, p65, p-IκB-a and GAPDH were obtained from Cell Signaling Technology. LightShift™ Chemiluminescent EMSA Kit was provided by Pierce Technology.

### Preparation of plant material and total saponins

Rhizome of *A. flaccida* Fr. Schmidt was collected from Jiufeng County of Hubei Province, China. TS isolated from *A. flaccida* were provided by Dr. Shiwei Jia (GKH Pharmaceutical Ltd.) and prepared as reported [13,15]. TS were dissolved in dimethyl sulfoxide (DMSO) and diluted in PBS for storage in −20°C freezer which is used in all subsequent experiments.

### Cell culture

RAW 264.7 cells were obtained from the American Type Culture Collection (ATCC, Manassas, VA). RAW 264.7 cells were grown in a-MEM supplemented with 10% heat inactivated FBS, 2 mM L-glutamine and 100 U/ml penicillin/streptomycin. Incubations were performed at 37°C in 5% CO2, and cultures were fed every 2–3 days.

### Cell survival viability assay

Cell viability was determined by 3-(4,5-dimethyl-2-thiazolyl)-2,5-diphenyl-2H-tetrazolium bromide (MTS) method using CellTiter 96® AQueous One Solution Cell Proliferation Assay (Promega, USA) according to the manufacturer’s instructions. The experiments were carried out 3 times in triplicate measurements.

### Osteoclast formation

RAW 264.7 cells were seeded onto a 96 well plate (1 × 10^4^ cells/well) with complete a-MEM containing RANKL (50 ng/mL) in the presence of TS or vehicle control for 6 days at 37°C and 5% CO_2_. After 6 days, cells were washed with PBS and fixed with 10% formaldehyde for 15 min. Then washed with PBS. Fixed cells were subjected to an assay for Tartrate-resistant acid phosphatase (TRAP) activity. The experiments were carried out 3 times in triplicate measurements.

### Preparation of Bone Marrow-derived Macrophages (BMMs) and in vitro osteoclast formation

Sprague -Dawley (SD) rats (4-7-week-old) were housed in Laboratory Animal Center (China Academy of Chinese Medical Sciences, Beijing, China) according to the guidelines for the care and use of laboratory animals of the NIH and China Academy of Chinese Medical Sciences (Beijing, China). All experimental procedures were approved by the Committee for Animal Use of the China Academy of Chinese Medical Sciences.

Total bone marrow cells were collected from tibia and femur of SD rat by flushing the marrow space with a-MEM. After removing the red blood cells (RBCs) with ACK buffer (0.01 mM EDTA, 0.011 M KHCO3, and 0.155 M NH4Cl, pH 7.3), cells were cultured for 1 day in a-MEM containing 10% fetal bovine serum (FBS). Nonadherent cells were collected and further cultured with 20 ng/mL M-CSF in a-MEM containing 10% FBS. After 3 days, culture medium was removed and adherent cells were used for osteoclast differentiation. BMMs were cultured for 6 days in medium containing 20 ng/mL M-CSF and 50 ng/mL RANKL to induce osteoclast formation.

### TRAP staining

TRAP staining kit was obtained from Sigma Alrich (USA). TRAP staining was carried out according to the manufacturer’s protocol. The images were taken with a digital camera attached to the microscope. TRAP positive multinucleated cells (≥3 nuclei) were scored as osteoclast-like (OCL) cells. The number of TRAP-positive cells was counted using an eyepiece graticule at a magnification of 100 and the results expressed as the number of cells per field of vision.

### Immunofluorescent staining

For F-actin ring staining, RAW 264.7 cells were stimulated with RANKL (50 ng/mL) for 6 days to induce osteoclast formation in the presence or absence of TS (0.1, 0.5 and 2.5 μg/mL). For NF-κB-p65 nuclear translocation experiment, RAW 264.7 cells were pretreated in the presence of TS or vehicle control overnight, then added RANKL (50 ng/mL) for 30 min. After incubation, the cultures were fixed in 4% paraformaldehyde in PBS and cells permeabilized with 0.1% Triton X-100 in PBS for 15 min. Cells were then stained for F-actin by incubation in 1 mg/ml TRITC-conjugated phalloidin or by anti-NF-κB-p65 for 2 h at 37°C. The preparations were then washed and mounted in Vectashield mounting medium with Hoechest 33258 (Vector Laboratories, Peterborough, UK). Cells were inspected with an Olympus x41 microscope. The experiments were carried out 3 times in triplicate measurements.

### Bone resorption pit assay

To study the effect of TS on osteoclastic bone resorption, RAW 264.7 cells were seeded onto 50 um thick bovine bone slices and cultured with complete a-MEM containing RANKL (50 ng/mL) in the presence of TS or vehicle control at 37°C and 5% CO_2_. After 7 days, cells were then removed by sonication and the bovine bone slices were stained with toluidine blue to identify resorption pits. Also resorption lacunae were visualized by a Hitachi S-3400 N scanning electron microscope.

### Enzyme Linked Immunosorbent Assay (ELISA)

RAW 264.7 cells were suspended in a-MEM containing 10% FBS and plated at a concentration of 1 × 10^5^ cells/well into a 24-well culture dish (Corning). Then, they were treated with either TS or vehicle for 24 h before adding RANKL (50 ng/mL) to stimulate TNF-a secretion for 16 h. At the end of culture, medium was collected and analyzed for TNF-a using the ELISA kit according to the manufacturer’s instructions.

### Western blotting

RAW 264.7 cells were seeded onto 75 cm^2^ flask pretreated with TS or vehicle control overnight at 37°C and 5% CO2. Then RANKL (50 ng/mL) was added for 30 min. Western blot was performed according to our previously described protocol [18]. The primary antibodies used in the experiment included anti-ERK1/2(1:1000), anti-phospho-ERK1/2 (1:200), anti-Jun N-terminal kinase (JNK) (1:200), anti-phospho-JNK(1:200), anti-p38(1:500), anti-phospho-p38(1:200), anti-IκBα-p (1:250), anti-c-Fos (1:1000), anti-TRAF6 (1:1000), anti-GAPDH (1:1000). Horseradish peroxidase (HRP)-conjugated anti-mouse and anti-rabbit antibodies (1:5000) were used as secondary antibodies. The membranes were then visualized using the ECL system (Pierce, USA). GAPDH (internal control) was used to confirm equal protein loading.

### Electrophoretic Mobility Shift Assay (EMSA)

RAW 264.7 cells were incubated with or without various concentrations of TS (0.1, 0.5, 2.5 μg/mL) for 12 h. The cells were then treated with RANKL (50 ng/mL) for 30 min. The nuclear protein extracted from the cells using a NE-PER Nuclear and Cytoplasmic Extraction Reagent Kit (Pierce, USA). EMSA were performed according to protocol of the LightShiftTM Chemiluminescent EMSA Kit. The synthetic biotinlabeled double-stranded NF-κB consensus oligonucleotide (5′-AGTTGAGGGGACTTTCCCAGGC-3′) were used to measure the effect of the TS on NF-κB nuclear protein–DNA binding activity. Briefly, nuclear proteins (30 μg), poly (dI-dC), and biotin-labeled double-stranded NF-κB oligonucleotide were mixed with the binding buffer. In addition, the excess amounts of unlabeled oligonucleotide were used for the competition assay to confirm specificity of binding. The DNA/protein complex was electrophoresed on 5% non-denaturing polyacrylamide gel. The DNA mobility shift due to binding of NF-κB complex was detected with enhanced chemiluminescence (ECL) reagent (Pierce, USA).

### Statistical analysis

All raw data were processed by authorized software SPSS 13.0. The data were analyzed by One-way ANOVA followed by Dunnett’s t-test to assess the statistical significance of the differences between the study groups. Differences were considered statistically significant when *P* was less than 0.05.

## Results

### TS suppresses RANKL-induced osteoclast differentiation

**Figure 1:**
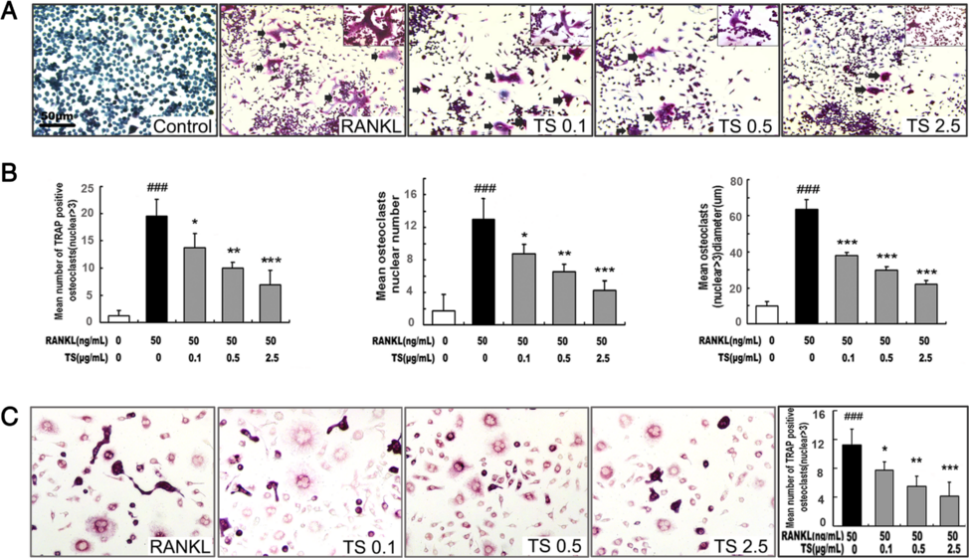
**TS inhibits RANKL-induced osteoclast differentiation. (A)** RAW 264.7 cells were cultured in the presence of RANKL with different concentrations of TS (0.1, 0.5 and 2.5 μg/mL). Six days post-culture, cells were fixed with 4% paraformadehyde followed by TRAP staining. Representative images of TRAP staining of OCLs from one of the three experiments are shown. **(B)** Quantitative analysis show the mean number of TRAP-positive OCLs, mean OCLs nuclei numbers, and mean OCLs sizes. **(C)** BMMs were cultured in the presence of RANKL with different concentrations of TS (0.1, 0.5 and 2.5 μg/mL). Six days post-culture, cells were fixed with 4% paraformadehyde followed by TRAP staining. All bar graphs represent mean ± SD of three independent experiments. ^###^ *P* < 0.001 significantly different from Control. *P < 0.05, **P < 0.01 and ***P < 0.001 significantly different from RANKL only group.

Since RAW 264.7 macrophage-like cells can differentiate into OCL cells as described elsewhere [19], the effect of TS on RANKL-induced osteoclast formation was examined using these cells. RAW 264.7 cells cultured in the presence of RANKL form multinucleated TRAP-positive OCL cells (Figure 1A). Addition of TS (0.1-2.5 μg/mL) into RAW 264.7 cells cultures showed dose-dependent decrease the number of TRAP positive multinucleated cells (Figure 1A and B). Notably, OCL cells in cultures that were treated with TS exhibited morphological differences with control OCL cells, being smaller in size and containing fewer numbers of nuclei (Figure 1A and B).

The effects of TS were further confirmed using primary BMMs. TS inhibited the multinucleated osteoclast formation induced by RANKL and M-CSF in BMMs (Figure 1C).

### TS attenuates RANKL-stimulated F-actin rings formation and osteoclastic bone resorption

**Figure 2:**
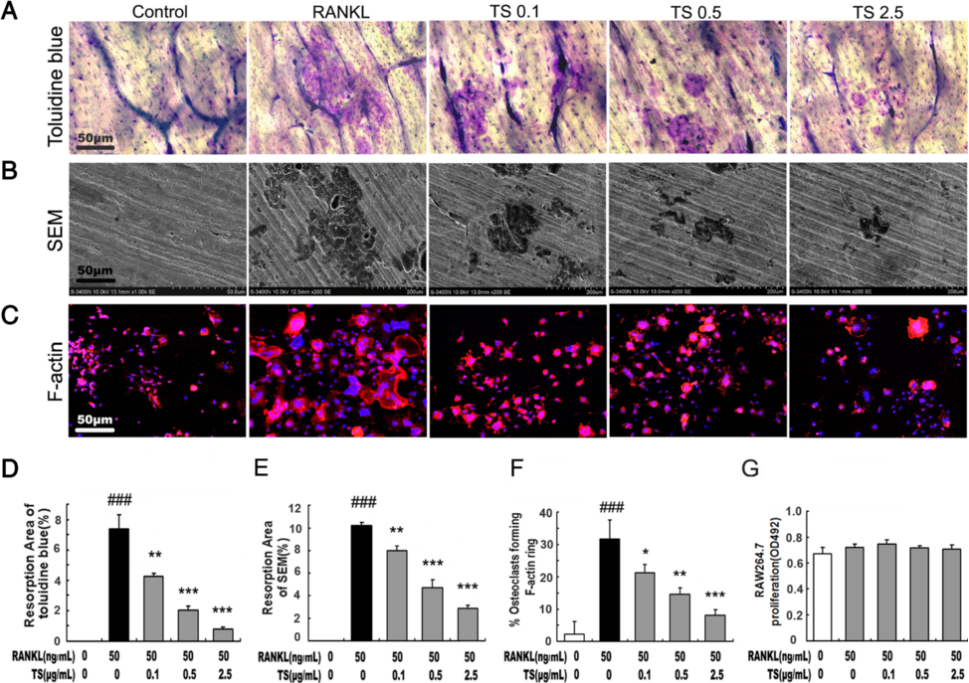
**TS abrogates osteoclast resorptive activity.** RAW 264.7 cell were cultured in the presence of RANKL with different concentrations of TS (0.1, 0.5 and 2.5 μg/mL). After 6 days, cells were removed, and the bone slices stained by toluidine blue **(A)** or by scanning electron microscopy (SEM) to identify resorption pits **(B)**. RAW 264.7 cells were cultured in the presence of RANKL with TS (0.1, 0.5 and 2.5 μg/mL). After 6 days of culture, cells were fixed with 4% paraformaldehyde and double stained with Honest 33258 (nuclear staining) and rhodamine-phalloidin (F-actin structure) and visualized by fluorescence microscopy **(C)**. Quantitative analysis shows the percentage of bovine bone slice surface occupied by resorption lacunae (**D** for toluidine blue staining and **E** for SEM), and percentage of osteoclasts forming F-actin rings **(F)**. The effects of TS on the viability of RAW 264.7 cells for 48 h were tested by MTS **(G)**. All bar graphs represent mean ± SD of three independent experiments. ^###^ *P* < 0.001 significantly different from Control. *P < 0.05, **P < 0.01 and ***P < 0.001 significantly different from RANKL only group.

To examine the effects of TS on osteoclastic bone resorption, equal numbers of RAW 264.7 cells were seeded onto bovine bone slices and thereafter TS was added to the culture. Bone surfaces were retrieved after incubation for 6 days, processed for toluidine blue staining or scanning electron microscopy, visualised and scored for bone resorption as described in the methods. Treatment of cultures with TS (0.1-2.5 μg/mL) can significantly attenuate osteoclastic bone resorption (Figure 2). Note that treatment of TS resulted in very shallower resorption pits or reduced pit areas as compared to untreated control both by toluidine blue staining or scanning electron microscopy (Figure 2A,B,D and E).

The bone resorption function of osteoclasts depends on dynamic regulation of the actin cytoskeleton. Actin ring structure is a characteristic cytoskeletal feature of functional osteoclasts [20]. Therefore, we next examined whether TS affects actin ring structure of mature osteoclasts. OCLs were double-stained with phallotoxins and Hochest 33258 to allow for visualization of the cytoskeleton and nuclei respectively. In mature osteoclasts on tissue culture plates, F-actin was arranged into a ring-like structure (actin ring) at the cell periphery. Treatment of with TS (0.1-2.5 μg/mL) caused both shrinkage of osteoclasts and disruption of actin ring structure in a dose-dependent manner (Figure 2C and F).

To examine whether the above suppressive effect of TS was due to its cytotoxicity, MTS assays were carried out to test cell survival viability. As shown in Figure 2G and Additional file 1: Figure S1, TS from 0.1 to 2.5 μg/mL did not shown any cytotoxicity on RAW 264.7 cells and BMMs under the experimental conditions used in the present studies. No cytotoxicity of TS was also confirmed by a trypan blue dye exclusion test (Additional file 2: Figure S2). Furthermore, apoptosis of RAW 264.7 was also unaffected by these anti-osteoclastic TS concentrations (Additional file 3: Figure S3), suggesting that the observed reduction of osteoclast formation and activity was a result of osteoclast differentiation inhibition by TS.

### TS inhibits RANKL-stimulated secretion of inflammatory cytokines

**Figure 3:**
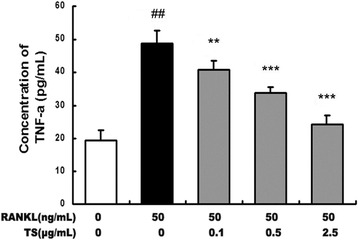
**TS inhibits RANKL-induced TNF-α s secreted by RAW 264.7 cells.** Confluent RAW 264.7 cells were incubated with or without TS (0.1, 0.5 and 2.5 μg/mL) for 2 h, followed by stimulation with RANKL (50 ng/mL) for 24 h, and supernatants were analyzed for TNF-α by enzyme-linked immunosorbnent assay (ELISA). Data are represented as the mean ± SD of three independent experiments. ^##^ *P* < 0.001 significantly different from control. **P < 0.01 and ***P < 0.001 significantly different from RANKL only group.

Inflammatory cytokines, such as TNF-a, induces osteoclasts formation, and stimulates osteoclastic bone resorption [21]. The effects of TS on the production of TNF-a induced by RANKL were studied on RAW 264.7 cells *in vitro*. As shown in Figure 3, RAW 264.7 cells produced a large quantity of TNF-a in response to RANKL stimulation. Treatment of RAW 264.7 cells with TS (0.1-2.5 μg/mL) effectively suppressed TNF-α production.

### TS abrogates RANKL-induced TRAF6 expression

**Figure 4:**
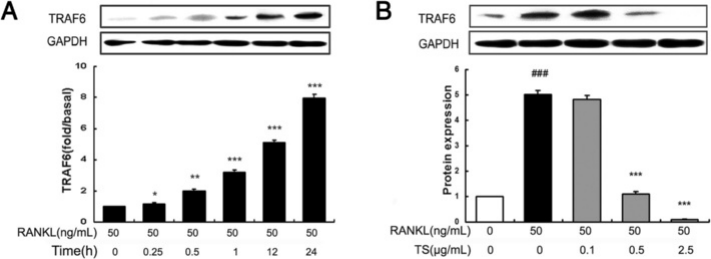
**TS abrogates expression of TRAF6 in RANKL-induced RAW 264.7 cells.** RAW 264.7 cells were pre-treated with RANKL (50 ng/mL), and cell lysates were collected at the time points indicated **(A)** and/or various concentrations of TS (0.1, 0.5 and 2.5 μg/mL) for 24 h. The harvested cells from triplicate tests were subjected to western blot analysis for TRAF6 **(B)**. Relative amount of protein were determined by densitometric analysis. One of three experiments with similar results is shown. Data are represented as the mean ± SD. ^###^ *P* < 0.001 significantly different from control. *P < 0.05, **P < 0.01 and ***P < 0.001 significantly different from RANKL only group.

The binding of RANKL with its receptor RANK interacts and induces the trimerization and activation of signaling adaptor molecule TRAF6, which is an essential initiating step during osteoclast differentiation [9]. We first analyzed the possible action of TS on TRAF6 protein expression by Western blot. As shown in Figure 4A, RANKL (50 ng/mL) stimulation obviously up-regulated the expression of TRAF6 in RAW 264.7 cells, reaching peak accumulation at 24 h. Therefore, in this study, we selected the induction time for 24 h to explore the effects of TS on TRAF6 protein expression. We found that TS (0.1, 0.5 and 2.5 μg/mL) pre-treatments markedly inhibited RANKL-stimulated expression of TRAF6 (Figure 4B).

### TS inhibits RANKL-induced MAPKs phosphorylation

**Figure 5:**
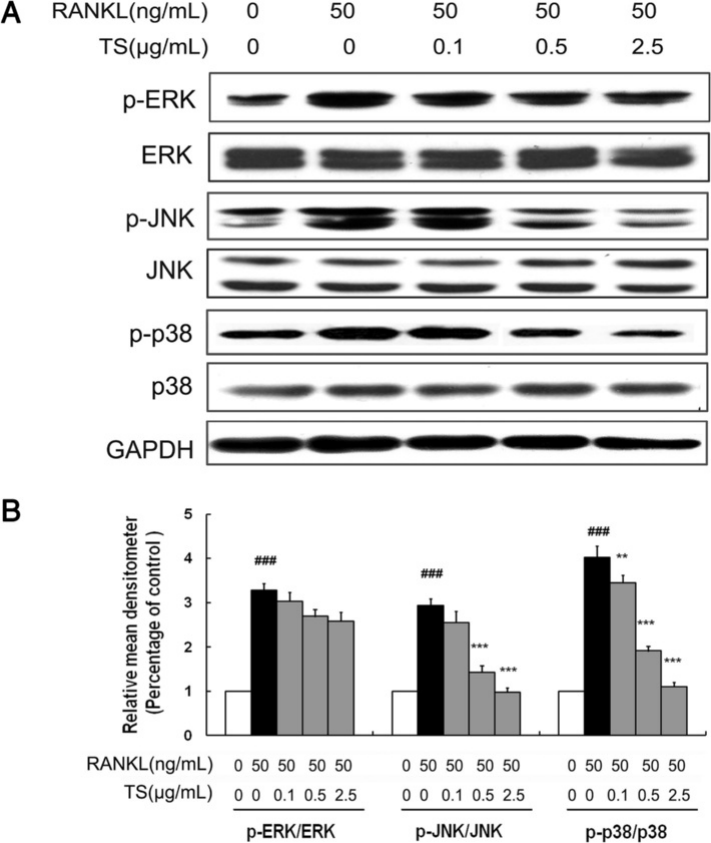
**TS attenuates JNK and p38 MAPKs activation in RANKL-induced RAW 264.7 cells.** RAW 264.7 cells were preincubated with the indicated concentrations of TS (0.1, 0.5 and 2.5 μg/mL) for 2 h, and then treated with 50 ng/mL RANKL for 30 min. Total protein was extracted and subjected to Western blot analysis **(A)**. For phosphorylated proteins, the ratio of phosphorylated to total species was determined by densitometry and normalization to glyceraldehyde 3-phosphate dehydrogenase (GAPDH) levels **(B)**. One of three experiments with similar results is shown. Data are represented as the mean ± SD three independent experiments. ^###^ *P* < 0.001 significantly different from control. **P < 0.01 and ***P < 0.001 significantly different from RANKL only group.

MAPKs (mainly including ERK, JNK and p38 MAPK) are located at the downstream of the TRAF6 signalling complexes, and play an important role in RANKL-induced osteoclast differentiation. To further explore pathways by which TS regulates osteoclast differentiation and function, the effect of TS on RANKL-induced ERK, JNK and p38 phosphorylation was examined in RAW 264.7 cells. RANKL strongly activated ERK, JNK and p38 phosphorylation in RAW 264.7, whereas RANKL-induced phosphorylation was inhibited by TS (0.1-2.5 μg/mL), most effectively for JNK, less for p38, and nearly no influence on ERK (Figure 5). These results indicated that the mechanism for the anti-osteoclast differentiation effects of TS involved the inhibition of JNK and p38 MAPKs.

### TS suppresses RANKL-induced Activation of NF-κB signaling pathway

**Figure 6:**
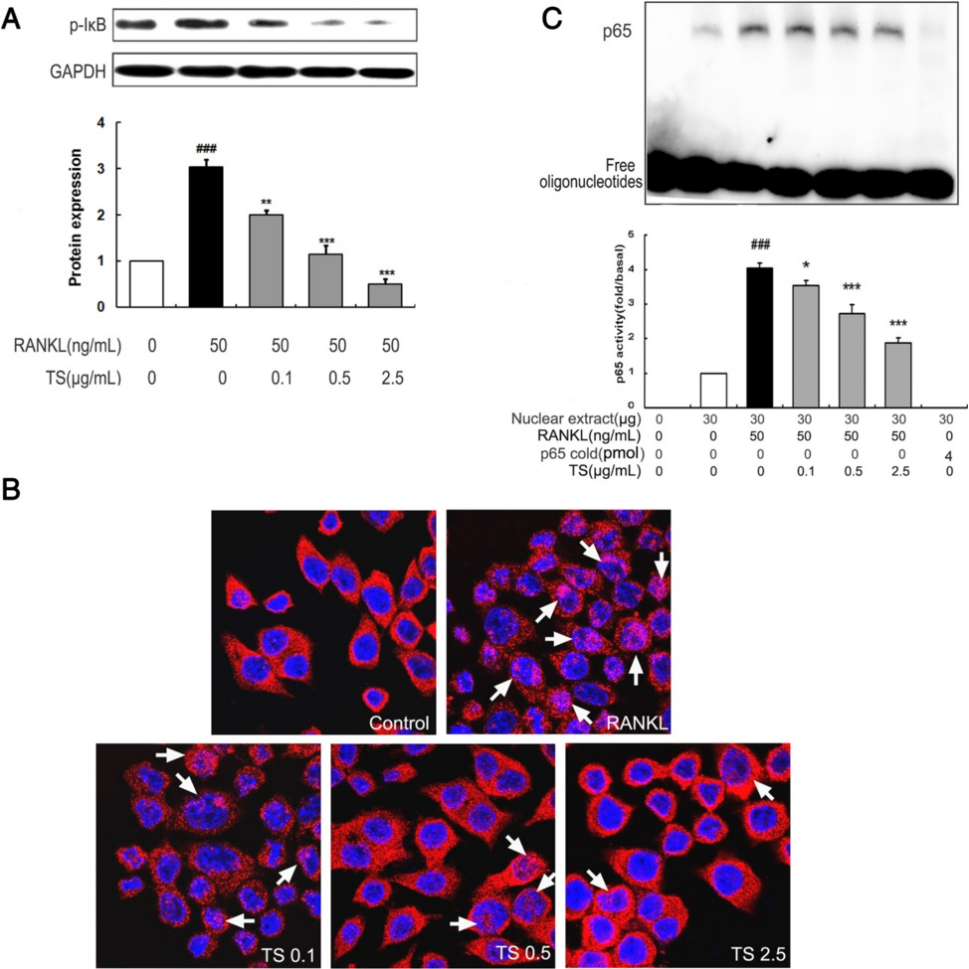
**TS suppresses RANKL-induced NF-κB activation in RAW 264.7 cells.** Confluent RAW 264.7 cells were pre-treated with various concentrations of TS (0.1-2.5 μg/mL) for 2 h followed by RANKL (50 ng/mL) for 30 min. The harvested cells from triplicate tests were subjected to western blot analysis for p-IκB-a. Relative amounts of protein were determined by densitometric analysis **(A)**. RAW 264.7 cells were pretreated with TS (0.1-2.5 μg/mL) overnight, followed by RANKL (50 ng/mL) for 30 min. NF-κB-p65 nuclear translocation analysis were performed by immunofluorescent staining **(B)**. RAW 264.7 cells were incubated with TS (0.1-2.5 μg/mL) for 12 h, followed by the addition of RANKL (50 ng/mL). After 30 min incubation, cells were analyzed for detection of DNA binding of NF-κB by EMSA. The arrowheads indicate the free probe and the specific DNA-probe/transcription factor complex (NF-κB), respectively. Relative NF-κB activity was calculated by densitometric analysis **(C)**. One of three experiments with similar results is shown. Data represent the mean ± SD of three independent experiments. ^###^ *P* < 0.001 significantly different from basal. *P < 0.05 and ***P < 0.001 significantly different from RANKL only group.

RANKL-induced NF-κB activation is essential in initiating osteoclast differentiation. To determine whether TS inhibited NF-κB-mediated osteoclast differentiation, we investigated NF-κB activation in RAW 264.7 cells affected by TS. As shown in Figure 6A, thirty minutes of RANKL (50 ng/mL) treatment alone, induced an increase in IκB-a phosphorylation, and addition of TS resulted in a marked suppression of IκB-a phosphorylation (Figure 6A). To add to this, we investigated the effect of TS on nuclear translocation of the NF-κB-p65 subunit in RAW 264.7 cells. Pre-treatment (2 h) of cells with TS (0.1, 0.5 and 2.5 μg/mL) prior to RANKL stimulation (0.5 h) resulted in a significant reduction in NF-κB-p65 nuclear translocation in RAW 264.7 cells, which was evidenced by the reduced amounts in the nucleus (Figure 6B). Further the NF-κB DNA binding activity was investigated in the activated RAW 264.7 cells. As shown in Figure 6C, the nuclear extract from RANKL-stimulated RAW 264.7 cells showed a marked increase in NF-κB nuclear protein DNA-binding activity compared with the control. The specificity of the NF-κB nuclear protein–DNA binding was verified by competition assay with a 200-fold excess of unlabeled NF-κB probe. Pretreatment of cells with various concentrations of TS suppressed the activation of NF-κB binding to its consensus DNA sequences. Taken together, these results indicated that the inhibition of the NF-κB pathway by TS contributed to its suppression of osteoclast formation.

### TS down-regulates RANKL-induced expression of NFATc1 and c-Fos

**Figure 7:**
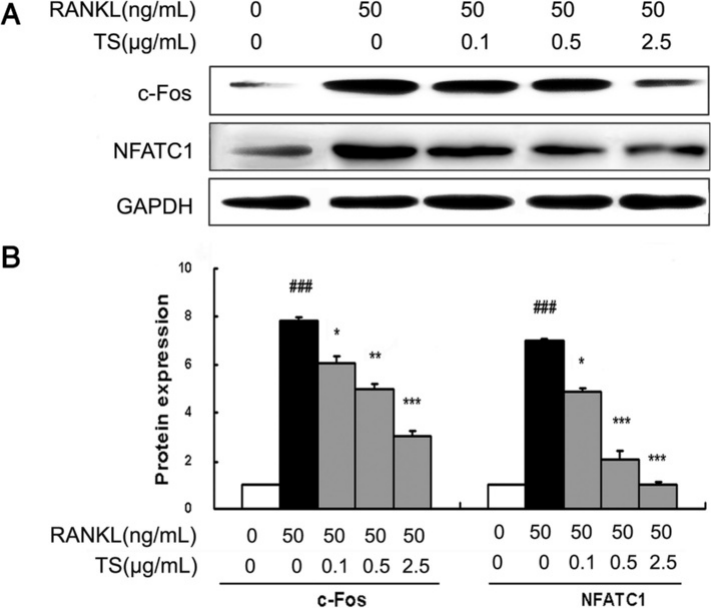
**TS down-regulates RANKL-induced expression of NFTAc1 and c-Fos in RAW 264.7 cells.** RAW 264.7 cells were preincubated with indicated concentrations of TS for 2 h, and then treated with 50 ng/mL RANKL for 24 h. Total protein was extracted and subjected to Western blot analysis using antibodies against NFATc1 and c-Fos **(A)**. Relative amounts of each protein were determined by densitometric analysis **(B)**. One of three experiments with similar results is shown. Data represent the mean ± SD of three independent experiments. ^###^ *P* < 0.001 significantly different from basal. *P < 0.05, **P < 0.01 and ***P < 0.001 significantly different from RANKL only group.

Binding of RANKL to RANK should activate several transcription factors that are responsible for promoting osteoclastic gene expression. NFATc1 and c-Fos are identified as two of the most important osteoclasts specific transcription factors [22]. As shown in Figure 7A and B, RANKL stimulation for 24 h obviously up-regulated the expression of both c-Fos and NFATc1 in RAW 264.7 cells. Additionally, TS (0.1, 0.5 and 2.5 μg/mL) treatments markedly inhibited the expression of c-Fos and NFATc1 with the concentration dependent manner.

## Discussion

The root of *A. flaccida* has long been used for the treatment of arthritis and traumatic injury in folk medicine of China. TS is the main active constituent of this herb medicine and has been previously demonstrated to exhibit beneficial effects on adjuvant-induced arthritis by its immunomodulatory and anti-inflammation effects [14,23]. However, the effects of TS on the bone resorption remain unclear. The results in this study showed that TS inhibits osteoclast formation and bone resorption via the suppression of RANKL-induced activation of NF-κB and MAPKs signaling pathway. These data provide the mechanistic explanation, at least in part for the protective effect of TS against lytic bone diseases, such as in RA.

Recent studies have identified osteoclasts as the principal cell type responsible for bone destruction [24]. Osteoclasts are unique multinuclear giant cells, which are derived from monocyte-macrophage lineage cells. Their differentiation from precursors can be induced by RANKL, a key factor that also controls the function and survival of mature osteoclasts [25]. As a ligand, RANKL interacts to its receptor RANK, and induces them to differentiate into osteoclasts. Notably, until now, no other endogenous factors that can lead to osteoclast formation without RANKL participation have been found. Therefore, RANKL is used as a convincing inducer in studies on differentiation and function of osteoclasts. In the present study, we adopted the RANKL-induced osteoclast differentiation platform from RAW 264.7 cells, and our results revealed that TS significantly reduced the osteoclast differentiation. It is generally accepted that the formation of bone resorption pits occurs in conjunction with the process of osteoclast differentiation. In addition, the results in our study showed that TS dramatically attenuates osteoclastic bone resorption as detected by bone resorption pit assay. These data confirmed that TS inhibits both the formation and the resorptive function of osteoclasts.

Inflammatory cytokines, such as TNF-a, induce pre-osteoclast fusion, support the survival of mature osteoclasts, and stimulate osteoclastic bone resorption [21]. It was recently reported that the RANK–RANKL pathway is involved in TNF-a-induced osteoclast differentiation [26-29]. Triterpenoid saponins from *A flaccida* have previously been reported to inhibit LPS-induced cyclooxygenase-2, and PGE2 expression in BEL-7402 and HepG2 hepatoma cell [15]. In the present study, TS strongly suppressed RANKL-induced TNF-a secretion by RAW 264.7 cells, which partly explains the application of TS in diseases with inflammatory bone destruction.

RANKL stimulates osteoclast precursors to commit to the osteoclastic phenotype by binding to its receptor RANK on the surface of osteoclast precursors [7]. The binding of RANKL with its receptor RANK interacts and induces the trimerization and activation of signaling adaptor molecule TRAF6, which is an essential initiating step during osteoclast differentiation [9]. Our data showed that TS significantly inhibited TRAF6 expression. As downstream of RANK signaling, three major subfamilies of MAPKs (ERK 1/2, JNK and p38 MAPK) have been implicated as key regulators of various cellular responses, including cell proliferation, differentiation and apoptosis [30,31]. These MAPKs also play pivotal roles in the development of osteoclasts [32-34], and are thus key molecular targets for therapeutic application in inflammatory bone diseases [35]. Indeed, specific inhibitors for ERK1/2, JNK and p38 MAPK could disturb RANKL-stimulated osteoclast differentiation [22,36-38]. In the present study, we demonstrated that TS could notably inhibit the RANKL-induced phosphorylation of JNK and p38 MAPK, suggesting that TS targets JNK and p38 MAPK cascades.

Genetic studies have demonstrated that NF-κB signaling pathway also plays a crucial role in osteoclast differentiation [11]. NF-κB knockout mice showed the defects of osteoclast differentiation and severe osteopetrosis, indicating that NF-κB is a crucial factor in osteoclast differentiation [39]. The classical NF-κB signaling pathway involves the activation of the IKK complex, which phosphorylates IκBα and targets them for ubiquitin-dependent degradation [9]. In the alternative IκB-independent pathway, direct phosphorylation of NF-κB subunit p65 by IKK also modulates NF-κB transcription activity [40]. In this study, TS inhibited RANKL-induced NF-κB activation by inhibiting p-IκBα and p65 nuclei translocation and DNA binding activity, which indicates that inhibiting the activation of NF-κB pathway contributed to the suppression of TS against osteoclast differentiation.

Following the activation of the MAPK and NF-κB signaling pathways caused by RANKL, multiple osteoclastogenic transcription factors, such as c-Fos, NFATc1 and PU.1, should be induced to mediate the formation of mature and functionally active osteoclasts [41]. When BMMs are stimulated by RANKL, the expression of NFATc1 is induced through c-Fos and auto amplification by NFATc1 [41,42]. NFATc1-deficient embryonic stem cells do not form mature osteoclasts by RANKL treatment and overexpression of ectopic ca-NFATc1 in BMMs appropriately induces osteoclast differentiation from BMMs even in the absence of RANKL [41,43]. c-Fos, a major component of the transcription factor AP-1, can induce the expression of NFATc1 to regulate osteoclast differentiation after RANKL stimulation. Mice lacking c-Fos develop osteopetrosis as a result of a complete ablation of osteoclast formation. Our results revealed that RANKL stimulation markedly up-regulated the expression of both c-Fos and NFATc1, which is dramatically suppressed by TS treatment.

## Conclusions

**Figure 8:**
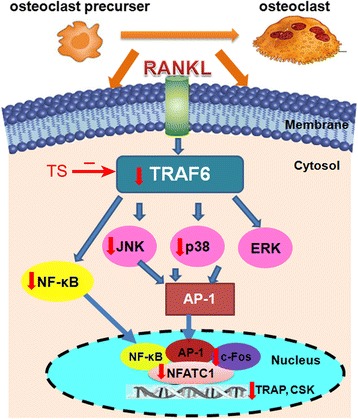
**Schematic model for TS regulation on the osteoclast differentiation and activation.** The figure summarizes the results presented with this study. Vertical arrows indicate that an inhibition or a downregulation in osteoclast was observed.

In conclusion, our results revealed for the first time that TS could suppress RANKL-induced osteoclast differentiation *in vitro*. We also clarified that the inhibitory effects of TS occur via the down-regulation of TRAF6 level, suppression of JNK, p38 MAPKs and NF-κB activation, subsequent decreased expression of c-Fos and NFATc1 (summarized in Figure 8). These results highlight the anti-osteoclast differentiation potential of TS, and provide further mechanism of TS in the application of RA. Furthermore, TS may be a potential agent and needs to be more evaluated *in vivo* or in clinical trials to become a therapeutic for the lytic bone diseases, especially inflammatory bone destruction.

## Additional files

Additional file 1: Figure S1. TS has no effect on BMMS viability. BMMs were treated with TS for 48 h, and cell viability was measured by a MTS viability assay. Data represent the mean ± SD of three independent experiments. Additional file 2: Figure S2. TS shows no effect on cell viability. RAW 264.7 cells were incubated with indicated concentrations of TS for 24 h, and then tested by trypan blue dye exclusion. Data represent the mean ± SD of three independent experiments. Additional file 3: Figure S3. TS treatment has no effect on cell apoptosis. RAW 264.7 cells were cultured in the presence of TS for 48 h and stained with Hoescht 33258, and analyzed with fluorescence microscope. Cells with nuclei containing condensed chromatin or cells with fragmented nuclei were defined as apoptotic cells. Magnification 600 × .
