# Correction to: Total saponin from *Anemone flaccida* Fr. Schmidt abrogates osteoclast differentiation and bone resorption via the inhibition of RANKL-induced NF-κB, JNK and p38 MAPKs activation

**DOI:** 10.1186/s12967-021-02868-5

**Published:** 2021-05-10

**Authors:** Xiangying Kong, Wenbin Wu, Yue Yang, Hongye Wan, Xiaomin Li, Michun Zhong, Hongyan Zhao, Xiaohui Su, Shiwei Jia, Dahong Ju, Na Lin

**Affiliations:** 1 Institute of Chinese Materia Medica, China Academy of Chinese Medical Sciences, No. 16, Nanxiaojie, Dongzhimennei, Beijing, 100700 China; 2 Institute of Basic Theory, China Academy of Chinese Medical Sciences, Beijing, 100700 China; 3 Guangzhou Kanghe Pharmaceutical Limited Company, Guangzhou, 511440 China

## Correction to: J Transl Med (2015) 13:91 https://doi.org/10.1186/s12967-015-0440-1

After publication of this article [1] it was brought to our attention that there were errors in Figs. 4a, b, 5a, 6a and 7a. We found that the GAPDH control bands were mistakenly placed. There were also some errors in the description of cell treatment in the legend of Fig. 4.

**Fig. 4:**
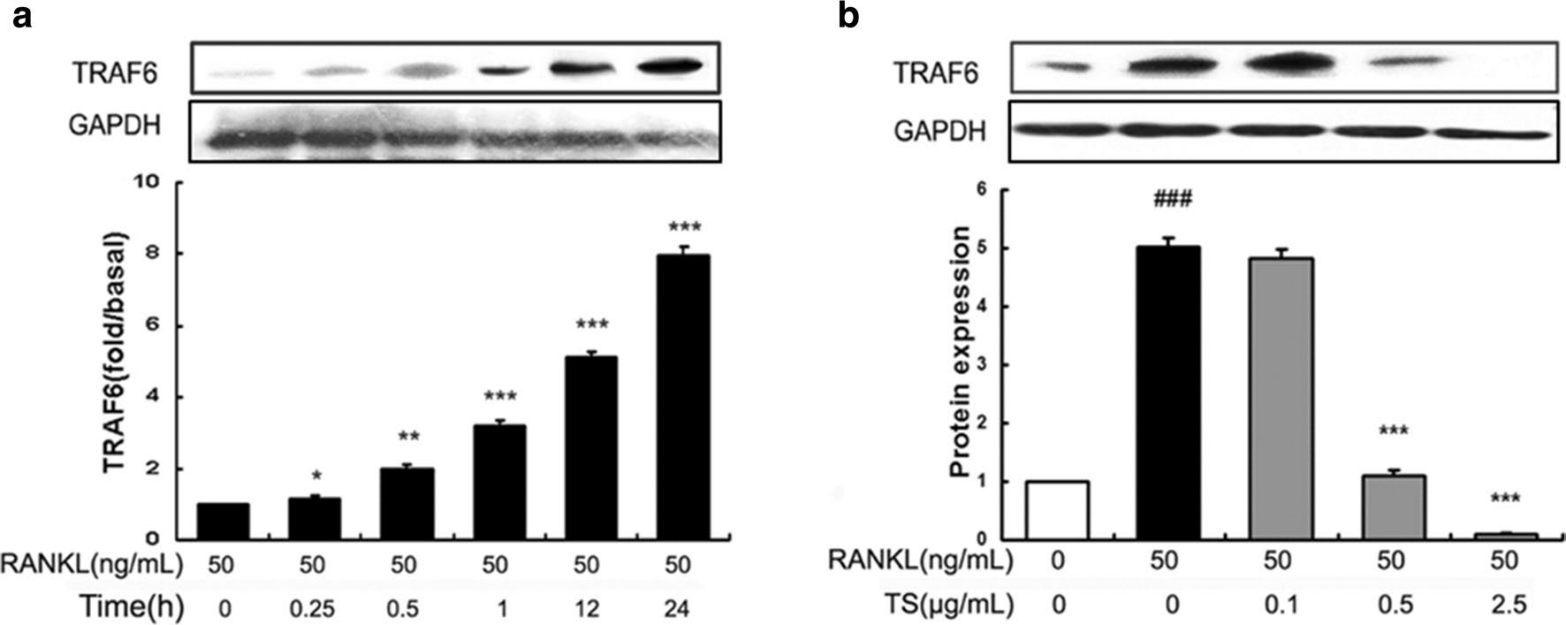
TS abrogates the expression of TRAF6 in RANKL-induced RAW 264.7 cells. **a** RAW 264.7 cells were treated with RANKL (50 ng/mL) and cell lysates were collected at the time points indicated. **b** RAW 264.7 cells were pre-treated with various concentrations of TS (0.1, 0.5 and 2.5 μg/mL) for 2 h, and then treated with 50 ng/mL RANKL for 24 h. The levels of TRAF6 in the cell extracts were determined by using western blot analysis. The relative amount of the protein was measured by densitometric analysis. The experiment was done three times under the same condition and the data are represented as the mean ± SD. ^###^*P* < 0.001 indicates that it is significantly different from control. *P < 0.05, **P < 0.01 and ***P < 0.001 indicate the significant difference from the RANKL only group.

 The corrected figures and figure legend are shown below. These corrections do not change the results or conclusions of the article. We apologise to readers for these errors.
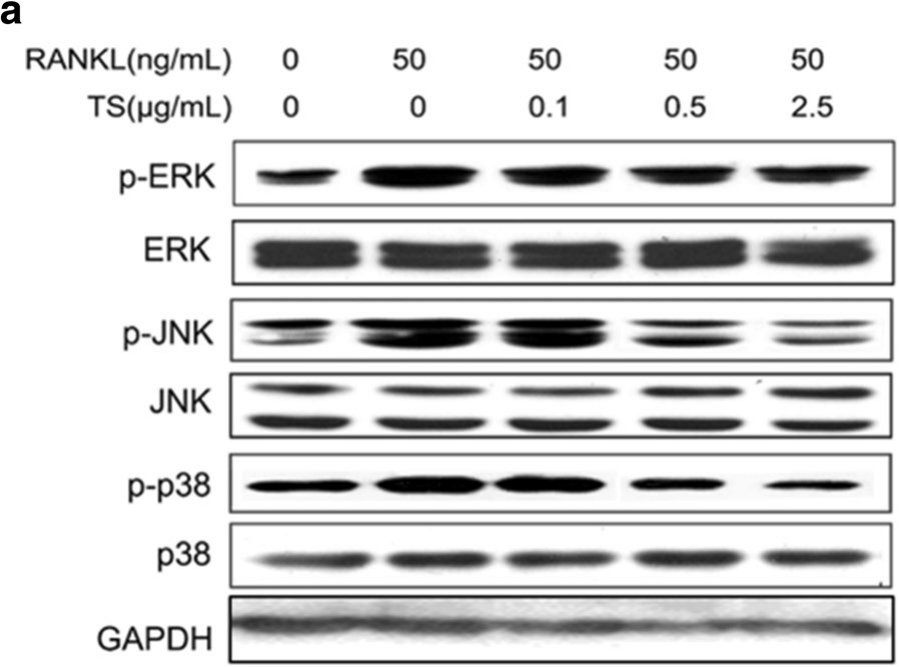


**Figure 5** The correct version of Fig. 5a. The full caption and figure are available via the original article.
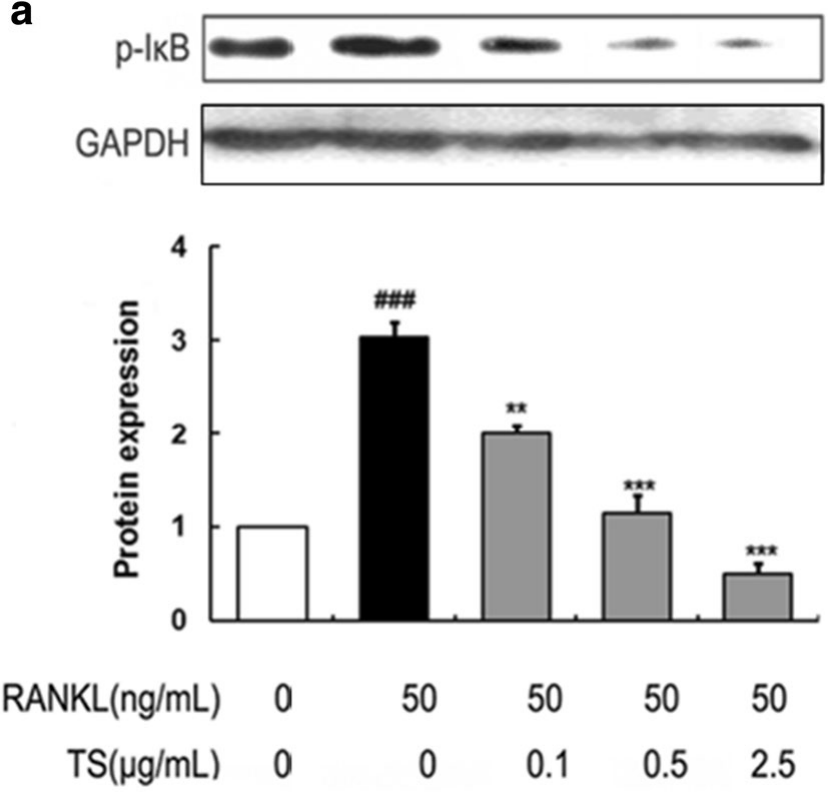


**Figure 6** The correct version of Fig. 6a. The full caption and figure are available via the original article.
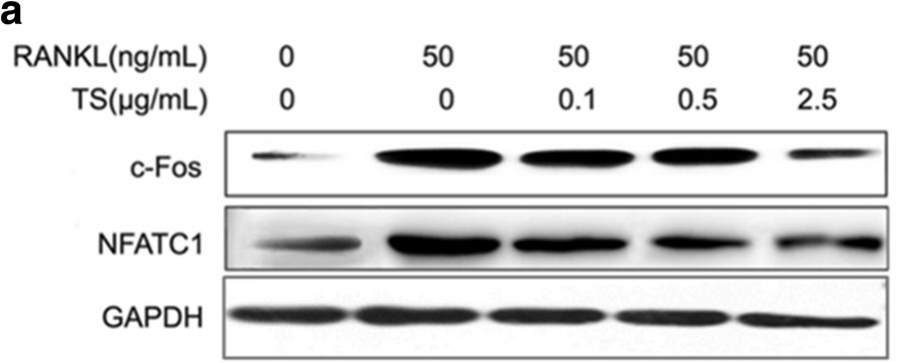


**Figure 7** The correct version of Fig. 7a. The full caption and figure are available via the original article.
