# Supplementary figures and images for: Total saponin from Anemone flaccida Fr. Schmidt abrogates osteoclast differentiation and bone resorption via the inhibition of RANKL-induced NF-κB, JNK and p38 MAPKs activation
Source: J Transl Med. 2015 Mar 15;13:91. doi: 10.1186/s12967-015-0440-1 (PMC4372222; doi:10.1186/s12967-015-0440-1)

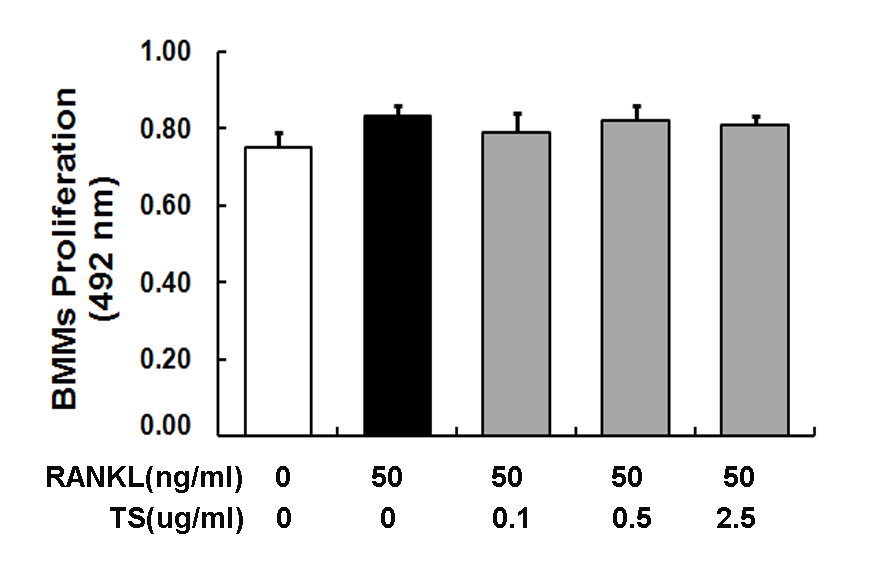

Supplement: Additional file 1: Figure S1. — TS has no effect on BMMS viability. BMMs were treated with TS for 48 h, and cell viability was measured by a MTS viability assay. Data represent the mean ± SD of three independent experiments. [file 12967_2015_440_MOESM1_ESM.tiff]

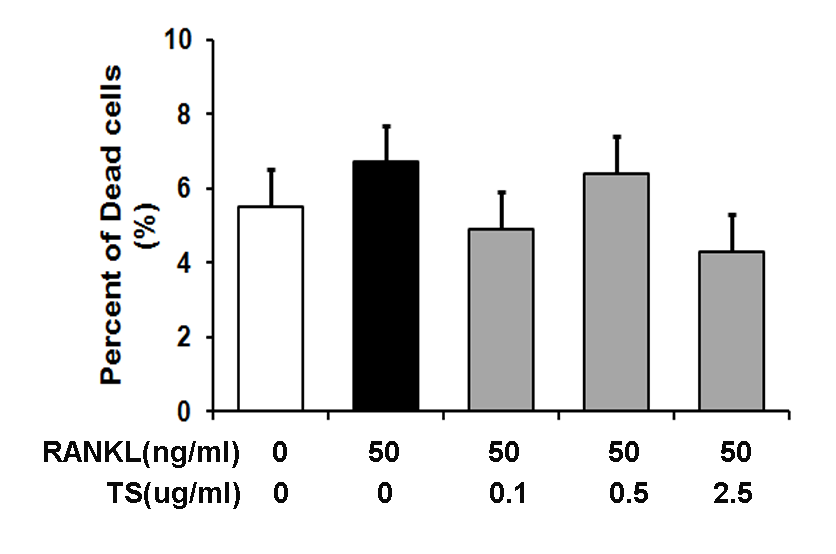

Supplement: Additional file 2: Figure S2. — TS shows no effect on cell viability. RAW 264.7 cells were incubated with indicated concentrations of TS for 24 h, and then tested by trypan blue dye exclusion. Data represent the mean ± SD of three independent experiments. [file 12967_2015_440_MOESM2_ESM.tiff]

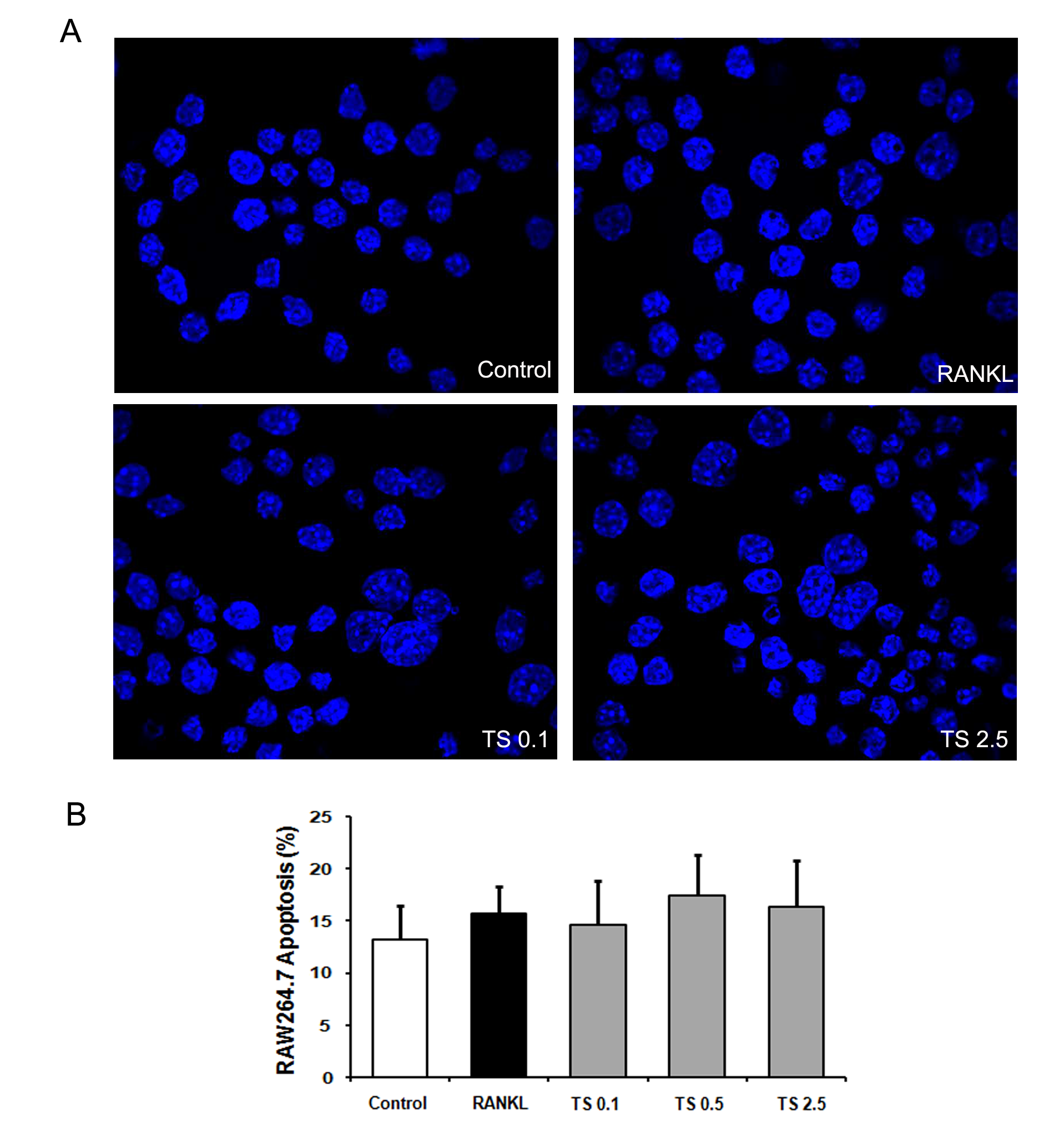

Supplement: Additional file 3: Figure S3. — TS treatment has no effect on cell apoptosis. RAW 264.7 cells were cultured in the presence of TS for 48 h and stained with Hoescht 33258, and analyzed with fluorescence microscope. Cells with nuclei containing condensed chromatin or cells with fragmented nuclei were defined as apoptotic cells. Magnification 600 × . [file 12967_2015_440_MOESM3_ESM.tiff]
